# Training of Resident Ophthalmologists in Cataract Surgery: A Comparative Study of Two Approaches

**DOI:** 10.1155/2015/932043

**Published:** 2015-05-14

**Authors:** Argyrios Tzamalis, Lampros Lamprogiannis, Nikolaos Chalvatzis, Chrysanthos Symeonidis, Stavros Dimitrakos, Ioannis Tsinopoulos

**Affiliations:** 2nd Department of Ophthalmology, Papageorgiou General Hospital, Faculty of Medicine, Aristotle University of Thessaloniki, 56403 Thessaloniki, Greece

## Abstract

*Purpose.* To evaluate and compare the efficacy of two different training methods in resident-performed phacoemulsification surgery.* Methods.* 502 eyes of 467 patients who underwent resident-performed phacoemulsification were included in the study by reviewing their medical records. Residents were allocated into two groups according to the method applied during their training in cataract surgery; Group A included residents that were trained with the “step-by-step” method and Group B those trained with the “one-step” method. Primary outcome was the incidence of main complications, defined as posterior capsular ruptures and/or zonular dehiscence with vitreous loss.* Results.* Each resident performed a median of 63 phacoemulsification surgeries. A statistically significant difference (*p* = 0.0032) was noted in the main complications rate between the two groups, yielding a mean of 17.3% in Group A and 7.25% in Group B. Other intraoperative complications were not shown to differ statistically significantly between study groups (*p* > 0.05). Among the first 40 surgeries of each resident, main complications rate differed also statistically significantly (*p* = 0.0048) between Group A (21.67%) and Group B (8.5%), while a better surgical performance-yielding statistical significance in Group A (*p* = 0.017) was indicated in both groups between the 20th and the 30th procedure.* Conclusions.* Training in cataract surgery using the “one-step” method may lead to an improvement in surgical competency, when measured by complications rates and, therefore, to significantly better quality of training for resident ophthalmologists.

## 1. Introduction

Age-related cataract is among the leading causes of severe visual impairment nowadays. With approximately 15 million cases performed annually, cataract extraction is one of the most frequent day-case procedures in medicine performed worldwide [1]. Anticipated demographic changes (population growth in developing countries and population ageing in both developing and developed countries) will gradually increase the amount of cataract-related visual morbidity and double the cataract subject pool in 20-year time [2].

This steadily increasing need for surgical services capable of delivering good quality vision rehabilitation leads to a subsequent need for qualified, proficient ophthalmic surgeons. To accommodate this situation, effective and validated surgical training programs are required to ensure that residents in training achieve greater levels of competence in cataract surgery. Wet laboratory training and simulators play an increasingly important role as part of an organized surgical curriculum [3, 4]. Surgical competency assessment tools are also being developed and integrated in ophthalmic training [5] and, in many residency programs, trainees' point of view of the quality of training they receive is investigated [6–8], as well as cataract surgery trainers' attitudes and techniques [9].

The learning curve for phacoemulsification cataract surgery is generally thought to be quite steep [10]. Posterior capsule rupture with or without vitreous loss is considered to be the main undesirable intraoperative complication of phacoemulsification surgery and in most studies complication rates as well as visual outcomes are used as indicators of success. As a matter of fact, the incidence of posterior capsule (PC) tear among trainee surgeons varies from 5.8% to 15% [10–12] and for PC tear with vitreous loss between 2.8% and 10% [13–16].

Phacoemulsification is often taught in stages, where a trainee performs a single part of the procedure several times in succession under the direct supervision of an experienced surgeon. When each step is mastered, the trainee then proceeds by learning the next step of the procedure. Other experienced tutors let residents try the whole procedure all at once. So far, a number of teaching techniques have been described [12, 17]; however, to the best of our knowledge, there are no published studies in the literature directly comparing their efficacy on resident surgical outcomes.

The aim of this study is to evaluate and compare the efficacy of two different training methods in resident-performed phacoemulsification surgery and assess their impact on residents' surgical training.

## 2. Materials and Methods

Five-hundred-and-two eyes of 467 patients, who underwent resident-performed cataract surgery by means of phacoemulsification at a tertiary care centre from 2008 to 2013, were included in a retrospective comparative study. The study was conducted in accordance with the tenets of the Declaration of Helsinki and was approved by the local institutional review board.

A retrospective analysis of all phacoemulsification cataract surgeries performed by ophthalmology residents from March 2008 to February 2013 in the 2nd Department of Ophthalmology, Aristotle University of Thessaloniki in “Papageorgiou” General Hospital, was conducted. Data were collected by reviewing patients' electronic medical records. Written informed consent to undergo cataract surgery was obtained from all patients and residents' participation in the operation was clearly disclosed. Operations performed by residents with previous surgical experience in other hospitals were excluded from this study. All surgical procedures were performed by 8 consecutive residents who did not have any previous experience as primary surgeon on intraocular procedures and completed at least 40 phacoemulsification surgeries.

Patients with cataract causing visual disturbance and no history of significant risk factors [18] were eligible for resident training. Additional exclusion criteria were ocular disease such as corneal opacity or irregularity, astigmatism greater than 2.5 D, severe dry eye syndrome, inadequate visualization of the fundus, IOL power calculation less than 10.0 diopters (D) or more than 30.0 D, surgical complications on the fellow eye, and incomplete follow-up. Patients with a history of uveitis and current intraocular inflammation, uncontrollable glaucoma, proliferative diabetic retinopathy, or retinal detachment were also excluded. Phacoemulsification was performed in all cases under topical anesthesia with eye drops of proparacaine hydrochloride ophthalmic solution 0.5% (Alcaine, Alcon Laboratories, CA, USA) using the Infiniti Vision System (Alcon Inc., TX, USA). All procedures were supervised by one high-volume attending surgeon (IT), who had the priority to take over in case of intraoperative complications or where the resident surgeon was unable to complete the procedure.

Residents were categorized in two groups, Group A and Group B, according to the method of surgical training they received. The allocation of residents to the training methods was conducted randomly and was not affected by unknown factors such as preferences of their supervisors. All residents received equal traditional didactic and wet-lab training prior to operating on patient eyes. Group A residents were trained with a step-by-step method, performing a gradually increasing number of phacoemulsification steps during the course of their training. Upon finishing each step successfully without any associated complications (at least 10 times), the resident was allowed to proceed by learning the next step of the procedure. Steps were taught in the same sequence as they appear in phacoemulsification surgery (corneal incisions; capsulorhexis; hydrodissection/hydrodelineation; phacoemulsification; irrigation/aspiration; IOL insertion/removal of viscoelastic device). Group B residents received a one-step method training program, during which they assisted in a minimum of five hundred (500) phacoemulsification operations and subsequently proceeded to perform the entire operation. All residents were trained to use “divide and conquer” phacoemulsification technique, which was considered easier to learn and safer for the patient.

The first 40 consecutive cases that each resident performed independently as the primary surgeon were additionally studied in a separate analysis. Outcomes of surgeries performed by Group A residents were compared with those of cases performed by their Group B counterparts. Primary outcomes were the incidence of main complications, defined as posterior capsular ruptures and zonular dehiscence with vitreous loss, and other intraoperative complications' rate.

Statistical analysis was performed with Medcalc statistical software (version 9.3.0.0, Medcalc, Mariakerke, Belgium) and SPSS (v. 17.0 for Windows, SPSS Inc., Chicago, IL). The data are given as mean ± standard deviation. Normality was checked using the Kolmogorov-Smirnov test. Due to the fact that data were not in all cases normally distributed, both parametric and nonparametric methods were used. The association of not normally distributed sizes was assessed through rank correlation calculating Spearman's coefficient rho. When parametric analysis was possible, the Student *t*-test was used to compare the outcomes between two groups. Categorical variables were compared using the Fisher exact probability test and chi-square test. Nonparametric Mann-Whitney *U* test was also used to examine associations between categorical variables and continuous or ordered outcomes. All *p* values were 2-sided and were considered statistically significant when less than 0.05.

## 3. Results

Eleven residents were trained during a 5-year time period between March 2008 and February 2013. Data from 3 residents were excluded from statistical analysis, 2 due to previous surgical experience on phacoemulsification in other ophthalmology departments and 1 due to short surgical education (23 completed phacoemulsification surgeries) not reaching the minimum surgical cases number (40). In total, data from 8 residents were enrolled in the retrospective analysis.

In total, the medical records of 502 phacoemulsification cases performed by 8 surgically naive residents were identified and reviewed. Each resident performed a median of 63 phacoemulsification procedures [range: 42–105]. All cases were allocated to two groups according to the method that was applied during the residents training in cataract surgery: Group A included cases from 3 residents that were trained with the “step-by-step” method and Group B included cases from 5 residents that were trained with the “one-step” method after assisting in at least 500 phacoemulsification cases performed by experienced instructors. No preoperative statistically significant difference was noted in the main demographics and clinical characteristics data between two groups, which are summarized in Table 1.

A statistically significant difference (*p* = 0.0032, Fisher's exact test) was noted in the main complications rate between the two groups, yielding a mean of 17.3% in Group A and 7.25% in Group B, respectively. Other intraoperative complications, such as anterior capsule tear or extended capsulorhexis (Group A = 4.86%, Group B = 4.42%), wound dehiscence (Group A = 1.62%, Group B = 0.95%), and corneal lesions of any kind (Group A = 0.54%, Group B = 0.32%), were not shown to differ statistically significantly between the two study groups (*p* > 0.05). A detailed list of all the complications with the percentages for each study group and the respective *p* values is provided in Table 2.

**Figure 1:**
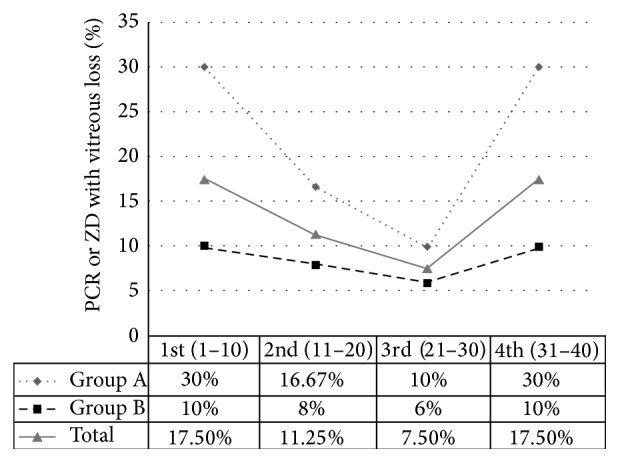
Incidence of posterior capsular rupture (PCR) or zonular dehiscence (ZD) with vitreous loss in each study group, dividing cases into decades as they were chronically performed by residents, referring to their first forty phacoemulsification procedures.

In order to minimize study bias originating from difference in the absolute number of procedures performed between the two study groups, a separate analysis was conducted including the first set of 40 consecutive cases performed by each resident, giving a total of 320 cases. Among these cases, capsular tear rates differed also statistically significantly (*p* = 0.0048, Fisher's exact test) between Group A (21.67%) and Group B (8.5%), while a better surgical performance, yielding statistical significance in Group A (*p* = 0.017, chi-square test) and not in Group B (*p* = 0.07, chi-square test), was indicated in both groups between the 20th and the 30th operation, where main complications rate was 7.5% in comparison to the chronologically 1st, 2nd, and 4th set of ten consecutive cases where this was assessed as 17.5%, 11.25%, and 17.5%, respectively. Complication rates for each study group and comparative data for the first 40 cases are shown in Table 3, whereas Figure 1 illustrates the percentage of posterior capsular tears in each group dividing cases into sets of 10 as they were chronologically performed.

**Figure 2:**
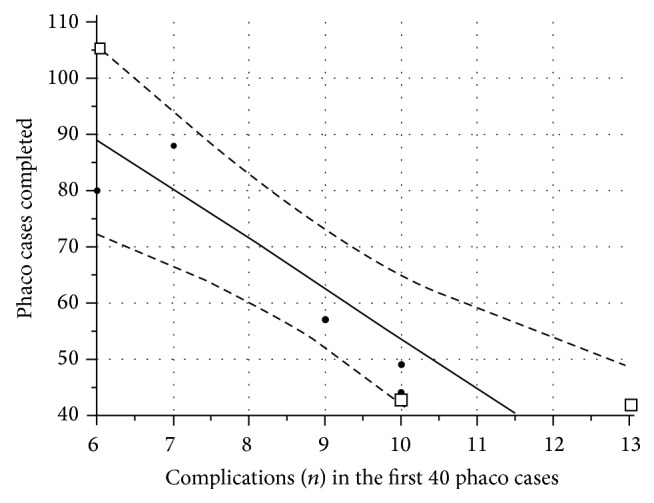
Scatter diagram and regression line (continuous line) showing correlation between the number of complications each resident had in their first 40 phaco cases and the total number of procedures that they were allowed to perform. The two dotted curves drawn parallel to the regression line represent a 95% confidence interval for the regression line. The residents of Group A are marked with white squares, whilst those of Group B are marked with black circles.

A statistically significant negative correlation (Spearman's *r* = −0.788, *p* = 0.035) was noted between the number of complications that residents faced in their first 40 surgical cases and the total number of procedures that each one was allowed to complete. The correlation diagram is shown in Figure 2.

## 4. Discussion

In a worldwide level, the demand for cataract surgery is expected to surge in the near future and this impending spike is multifactorial. Aside from a sharp increase in the number of aging patients with cataracts, evolving surgical techniques and better surgical outcomes promise to boost demand among relatively young, active patients who desire early cataract surgery or refractive lens exchange. The key solution to accommodate this situation is to enhance the efficiency and safety in current phacoemulsification training programs in order to generate better qualified cataract surgeons.

A variety of teaching methods and modalities have been described on phacoemulsification training [3, 4, 7, 9, 12–15]. Traditional programs include studying basic literature on phacoemulsification, wet-lab practice, and starting phacoemulsification under supervision [9, 14]. Another basic part of training in phacoemulsification is assisting in cases of experienced tutors [18]. Acting as a scrubbing assistant for more than several hundred cases offers candidates the ability to collect useful ideas pertaining to basic phacoemulsification processes, risk evaluation, and recognition of early signs of complications and their management [19].

Most phacoemulsification trainers fear that moving directly to resident-performed surgery would lead to higher complication rates. As a matter of fact, many phaco tutors tend to allow an assistant to perform only some simple steps of the procedure, such as irrigation-aspiration and insertion of an IOL, following successful practice on porcine eyes. To the best of our knowledge though, there are no published studies directly comparing the efficacy of step-by-step method to one-step method in terms of intraoperative complications rate.

Therefore, in our study, we attempted to assess and compare the efficacy of the above mentioned different training methods in resident-performed phacoemulsification surgery. Our results show that residents trained with the one-step method outperformed Group A residents (step-by-step teaching program), as shown by lower percentages of main intraoperative complications, defined as posterior capsular ruptures and zonular dehiscence with vitreous loss. A statistically significant difference was noted in these complications' rate between the two groups, yielding a mean of 17.3% in step-by-step group and 7.25% in one-step group. Taking into account only the first forty cases per resident the incidence of main complications was increased to 21.67% and 8.5% in the above mentioned groups, respectively, and PCR rate was 17.5% and 6%, respectively. Yulan et al. in a recently published study on step-by-step phacoemulsification training program for ophthalmology residents found lower posterior capsular rates (10.8 ± 4.2%) in the first 30 cases performed by each trainee [19].

There is a great variety of studies assessing the incidence of intraoperative complications in resident phacoemulsification surgeries. Hashemi et al. demonstrated that, among 500 cases operated by residents, 51 (10.2%) developed vitreous loss and 48 (9.6%) developed posterior capsule rupture and vitreous loss [20]. They concluded that direct attending supervision and careful case selection for the level of cataract surgery residency were of utmost importance in avoiding sight-threatening complications. Pokroy et al., trying to determine whether virtual surgery simulator training improves actual resident cataract surgery performance, found that capsular tear rates for the nonsimulator and simulator groups were 8.8% and 10%, respectively, for the first 25 cases [21]. Carricondo et al., studying resident-performed phacoemulsification surgery, indicated a statistically significant difference in PCR rates between the first 40 cases per resident and the total number of operations, yielding a mean of 9.65% in the first 40 operations, data comparable to our results [22].

Several other studies showed lower complications rates in residents training programs varying from 3% to 7% [23–27]. This difference may be attributed to the fact that residents included in these studies were not in all cases surgically naive and the complications rate was calculated for a larger amount of phacoemulsification cases performed by each resident, which could possibly lower the cumulative complications rate according to the learning curve.

The superior performance of residents trained with the one-step method may be attributed to a better perception of cataract surgery. Young ophthalmic surgeons are taught that an operation does not consist of a number of independent steps and they realize that every mistake, even the slightest, leads to increased difficulty and complication rates. Findings of the present study indicate that integration of the one-step method in ophthalmology training programs may lead to fewer complications and, therefore, to improved surgical outcomes. Obstacles such as the necessity of a long period of assistance in operations performed by experienced surgeons and trainers' possible hesitation to allow a surgeon to undertake an operation from beginning to completion need to be overcome. Incorporation of the one-step method in resident training programs, as it has been demonstrated, may improve the quality of ophthalmology residency training and accommodate the increasing demand for skilled ophthalmic surgeons.

Another major finding of the present study is that residents, during their first 40 operations, showed a better performance in terms of lower intraoperative complications rate in the 3rd set of 10 cases where main complications rate was 7.5% in comparison to 1st, 2nd, and 4th set where this was assessed as 17.5%, 11.25%, and 17.5%, respectively. The above mentioned fluctuation in complications rate according to level of experience was noticed in both groups, independently of the training method used. We believe this interesting remark may be attributed to the fact that most residents neglect several important rules once they feel confident enough to reduce operating times after their first 30 cases.

The amount of phacoemulsification cases performed exclusively by an ophthalmology resident, or the completion rate, should also be considered as another critical aspect in resident surgical training. Every supervisor should prevent some potentially dangerous or inappropriate movements and take over the case if serious intraoperative complications occurred. Supervisor's attitude and alertness during residents' operating attempts somewhat modify the incidence of complications and only this parameter could not reflect the real acquisition of skills in phacoemulsification [28]. A phacoemulsification trainer should strive to give equal support and opportunities to each resident during their training program [9, 16]. However, in a patient-based practice, most supervisors will try their best to avoid a high complication rate. This common belief is in concordance with our finding that residents with lower complications rates in their first 40 cases were allowed to perform significantly more phacoemulsification surgeries during their residency programs.

Limitations of our study include its retrospective design and the review of a single attending physician's technique. However, consistency in technique and instrumentation was important to analyze the impact of different teaching methods on residency cataract surgery training. In addition, confounding data, such as ocular comorbidities, cataract characteristics of patients, and other possible risk factors, were not accounted for in this study. Furthermore, although surgical cases per resident as a mean did not differ statistically significantly between the two groups, there was a quite uneven distribution of performed phaco cases among trainees, ranging from 42 to 105 surgeries per resident. Based on the analysis, this was mainly attributed to the retrospective nature of the study and to the fact that residents with more complications had fewer surgical opportunities after the first 40 cases. This finding could affect the overall complication rate and should be considered.

In conclusion, to the best of our knowledge, this study is the first to compare directly the “step-by-step” to the “one-step” teaching methods in phacoemulsification training of residents. The results of our study imply the fact that training in cataract surgery with the use of “one-step” method may enhance surgical performance, when measured by intraoperative complication rates, and, therefore, lead to a better quality of training for resident ophthalmologists, improving their learning curve. Further investigation analyzing the effect of various methods of resident surgical training on final corrected visual acuity is warranted in the future.

## Figures and Tables

**Table 1:** Main demographic and clinical characteristics data.

	Group A	Group B	*p* value	Total
Phaco cases	185	317		502
[per resident]	[61.67 ± 27.6]	[63.4 ± 19.7]	0.93	
Gender	88F/75M	161F/143M	0.87	249F/218M
Age (years)	73.4 ± 10.9	72.8 ± 9.2	0.53	73.1 ± 10.5
Axial length (mm)	23.8 ± 3.7	23.6 ± 4.2	0.59	23.7 ± 4.1
Preoperative MRSE	0.39 ± 1.4	0.45 ± 1.5	0.68	0.42 ± 1.5
Postoperative MRSE	−0.32 ± 0.2	−0.35 ± 0.2	0.11	−0.34 ± 0.4
IOL power	21.3 ± 2.7	21.6 ± 2.9	0.25	21.5 ± 2.9

MRSE = manifest refractive spherical equivalent; IOL = intraocular lens.

Student's *t*-test was used for comparing data between Group A and Group B in the following parameters: age, axial length, and IOL power.

Mann-Whitney *U* test was used for comparing data between Group A and Group B in the following parameters: phaco cases per resident, preoperative MRSE, and postoperative MRSE.

Fisher's exact test was used for comparing data between Group A and Group B in the following parameter: gender.

**Table 2:** Intraoperative complications rates of all study cases for each study group and respective *p* values. Percentages are given in parentheses.

	Group A	Group B	*p* value^†^	Total
PCR without lens fragment fall	26 (14.05%)	16 (5.05%)	0.0026	42 (8.36%)
ZD	2 (1.08%)	3 (0.95%)	1	5 (1%)
PCR with lens fragment fall	5 (2.7%)	6 (1.89%)	0.55	11 (2.19%)
PCR/ZD with vitreous loss	**32** (**17.3%**)	**23** (**7.25%**)	**0.0032**	**55** (**10.95%**)
ACT/EC	9 (4.86%)	14 (4.42%)	0.83	23 (4.58%)
WD	3 (1.62%)	3 (0.95%)	0.67	6 (1.19%)
CL	1 (0.54%)	1 (0.32%)	1	2 (0.4%)
ACRLF	2 (1.08%)	3 (0.95%)	1	5 (1%)

PCR = posterior capsule rupture; ZD = zonular dehiscence; ACT = anterior capsule tear; EC = extended capsulorhexis; WD = wound dehiscence; CL = corneal lesions; ACRLF = anterior chamber retained lens fragments.

^†^ *p* value (significance level) was calculated by means of Fisher's exact test.

**Table 3:** Intraoperative complications rates of the first 40 phacoemulsification cases per resident for each study group and respective *p* values. Percentages are given in parentheses.

	Group A	Group B	*p* value^†^	Total
PCR without lens fragment fall	21 (17.5%)	12 (6%)	0.0047	33 (10.31%)
ZD	1 (0.83%)	2 (1%)	1	3 (0.94%)
PCR with lens fragment fall	5 (4.16%)	3 (1.5%)	0.27	8 (2.5%)
PCR/ZD with vitreous loss	**26** (**21.67%**)	**17** (**8.5%**)	**0.0048**	**43** (**13.43%**)
ACT/EC	5 (4.17%)	11 (5.5%)	0.79	16 (5%)
WD	3 (2.5%)	3 (1.5%)	0.68	6 (1.87%)
CL	1 (0.83%)	1 (0.5%)	1	2 (0.62%)
ACRLF	2 (1.67%)	3 (1.5%)	1	5 (1.56%)

PCR = posterior capsule rupture; ZD = zonular dehiscence; ACT = anterior capsule tear; EC = extended capsulorhexis; WD = wound dehiscence; CL = corneal lesions; ACRLF = anterior chamber retained lens fragments.

^†^ *p* value (significance level) was calculated by means of Fisher's exact test.
